# Methylation-capture and Next-Generation Sequencing of free circulating DNA from human plasma

**DOI:** 10.1186/1471-2164-15-476

**Published:** 2014-06-15

**Authors:** Kristina Warton, Vita Lin, Tina Navin, Nicola J Armstrong, Warren Kaplan, Kevin Ying, Brian Gloss, Helena Mangs, Shalima S Nair, Neville F Hacker, Robert L Sutherland, Susan J Clark, Goli Samimi

**Affiliations:** Garvan Institute and The Kinghorn Cancer Centre, 370 Victoria Street, Darlinghurst, Sydney NSW 2010 Australia; School of Mathematics and Statistics, University of Sydney, Sydney, NSW 2006 Australia; Centre for Clinical Genomics, Garvan Institute and The Kinghorn Cancer Centre, Sydney, NSW 2010 Australia; The Ramaciotti Centre for Gene Function Analysis, University of New South Wales, Sydney, NSW 2052 Australia; School of Women’s and Children’s Health, University of New South Wales, and Gynaecological Cancer Centre, Royal Hospital for Women, Sydney, NSW 2052 Australia; St. Vincent’s Clinical School, Sydney, University of New South Wales, Sydney, NSW 2052 Australia

**Keywords:** Free circulating DNA, Next-Generation Sequencing, Methylation, Biobanking, Blood

## Abstract

**Background:**

Free circulating DNA (fcDNA) has many potential clinical applications, due to the non-invasive way in which it is collected. However, because of the low concentration of fcDNA in blood, genome-wide analysis carries many technical challenges that must be overcome before fcDNA studies can reach their full potential. There are currently no definitive standards for fcDNA collection, processing and whole-genome sequencing. We report novel detailed methodology for the capture of high-quality methylated fcDNA, library preparation and downstream genome-wide Next-Generation Sequencing. We also describe the effects of sample storage, processing and scaling on fcDNA recovery and quality.

**Results:**

Use of serum *versus* plasma, and storage of blood prior to separation resulted in genomic DNA contamination, likely due to leukocyte lysis. Methylated fcDNA fragments were isolated from 5 donors using a methyl-binding protein-based protocol and appear as a discrete band of ~180 bases. This discrete band allows minimal sample loss at the size restriction step in library preparation for Next-Generation Sequencing, allowing for high-quality sequencing from minimal amounts of fcDNA. Following sequencing, we obtained 37×10^6^-86×10^6^ unique mappable reads, representing more than 50% of total mappable reads. The methylation status of 9 genomic regions as determined by DNA capture and sequencing was independently validated by clonal bisulphite sequencing.

**Conclusions:**

Our optimized methods provide high-quality methylated fcDNA suitable for whole-genome sequencing, and allow good library complexity and accurate sequencing, despite using less than half of the recommended minimum input DNA.

**Electronic supplementary material:**

The online version of this article (doi:10.1186/1471-2164-15-476) contains supplementary material, which is available to authorized users.

## Background

Free circulating DNA (fcDNA) is DNA found in blood, not associated with any cell fraction, and occurs predominantly as fragments of approximately 180 bases with a smaller proportion of 360 base fragments [1, 2]. The size suggests that it originates from apoptotic cells, as it corresponds to the length of DNA wrapped around mono- and di-nucleosomes, and to the two smallest bands of the apoptotic DNA ladder, consistent with DNA cleaved at inter-nucleosomal sites. The apoptotic origin is further supported by the observation that fcDNA is increased in mouse plasma when liver apoptosis is induced by administration of anti-CD95 antibody [1]. Mice injected with anti-CD95 antibody showed an increase in fcDNA, in parallel with the appearance of the characteristic mono- and di-nucleosome sized bands on a DNA gel. In contrast, mice in which liver necrosis was induced by acetaminophen also showed an increase in fcDNA; however this DNA was of high molecular weight, and no increase in mono- or di-nucleosome bands was apparent. These data support the apoptotic rather than necrotic origin of fcDNA.

The cell type which contributes most to the fcDNA in healthy subjects is unclear. Measurement of Y-chromosome DNA in the plasma of female patients receiving bone marrow from male donors showed that the DNA is predominantly of donor origin, hence derived from hematopoietic cells [3]. However, another group excluded T-cells as a source of fcDNA based on lack of T-cell specific sequence rearrangement [1]. fcDNA derived from cells other than T-cells was measured using primers designed to amplify the germline configuration of T-cell receptor β-chain genes and T-cell receptor DNA was measured using primers designed to amplify the rearranged T-cell receptor β-genes found in mature T-cells. All fcDNA samples contained the germline sequence of T-cell receptor β-chain genes, while 18 of the 20 cancer fcDNA samples tested showed no amplification with the T-cell specific primers, indicating that T-cells are not a major source of fcDNA in cancer patients.

Due to the non-invasive nature by which fcDNA can be collected and examined, it carries tremendous potential in clinical applications. One of the earlier clinical applications of fcDNA is for non-invasive pre-natal diagnosis. Fetal DNA can be detected in the maternal circulation starting from about the 10^th^ week of pregnancy [4], and quantitation can accurately identify aneuploidies [5] without the increased risk of miscarriage associated with more direct sampling of fetus-derived tissues. Differences in DNA methylation have been used to discriminate between fetal and maternal DNA within the fcDNA pool [6, 7]. These differences may allow fetal DNA to be distinguished from the large background of maternal DNA, and permit a more accurate identification of fetus-specific DNA changes.

More recently, studies have demonstrated that fcDNA levels are increased in cancer patients as a result of tumor cells shedding DNA into the blood (recently reviewed in [8] and [9]), suggesting that fcDNA may be useful for cancer detection. Moreover, studies have demonstrated that cancer-derived fcDNA carries the same molecular aberrations, including mutations and methylation changes, as the source tumor, suggesting its value as a cancer biomarker. For example, *KRAS* mutations in fcDNA matching those in the solid tumor have been detected in pancreatic cancer [10], colorectal cancer [11], and lung cancer [12], while mutated *BRAF* sequences have been detected in the fcDNA of melanoma patients [13]. Jahr *et al*. showed that *CDKN2A* promoter methylation was present in fcDNA and corresponding solid tumors in 44% of cases examined, and absent from healthy controls [1]. It has been shown that the presence of colorectal and breast tumours can be determined from the quantity of chromosomally aberrant DNA in the circulation, without reference to specific individual mutations [14]. In addition to detecting the presence of a tumor, fcDNA is potentially a clinically useful tool for characterizing heterogeneous patient subtypes and for monitoring response to therapy [13]. Hence there is an interest in the molecular characterization in fcDNA of cancer patients in order to identify biomarkers for diagnosing the disease, determining tumor subtypes, and tracking chemo-response.

Recent advances in whole-genome sequencing have propelled our understanding of the germline and somatic genomic alterations that are associated with cancer development and progression (reviewed in [15]). Despite the many benefits and clinical applications of fcDNA, whole-genome analysis presents a number of technical challenges, particularly because in healthy individuals total fcDNA is present at low concentrations (typically 1–27 ng/ml) [8]. There are currently no definitive standards for fcDNA collection, processing and whole-genome sequencing and existing protocols do not allow straightforward Next-Generation Sequencing (NGS) analysis of the methylated fraction of fcDNA. While affinity purification and parallel sequencing of methylated DNA perform robustly in samples where abundant starting material is available [16], the limitations of these techniques in plasma samples are two-fold: first, fcDNA occurs at a very low concentration in control subjects, and this makes selective binding of the methylated DNA fraction difficult, as non-specific binding dominates the captured sample; secondly, the methylation enrichment step only recovers around 7% of the total DNA input [17, 18], which dramatically reduces the amount of DNA available for NGS library construction and sequencing. Hence, relatively large volumes of blood are required in order to purify sufficient quantities of methylated fcDNA to be compatible with downstream Next-Generation Sequencing.

In an effort to address and resolve these technical challenges, we report our comprehensive technical analysis of fcDNA isolation from healthy subjects and enrichment of methylated sequences followed by Next-Generation Sequencing. We describe a purification process optimized for use with very dilute samples, methylation sequence enrichment from low quantities of input DNA, and the library quality and read numbers derived from these samples. Our protocols allow for processing and high-quality genomic methylation analysis from as little as 50 ng of total fcDNA, including library preparation from less than half of the recommended minimum input material.

## Methods

### Clinical sample collection

Blood collection from consented volunteers was approved by the Human Research Ethics Committee at St Vincent’s Hospital (HREC 09/100). For plasma separation, blood was collected in 10 mL Vacutainer plastic tubes which contain K_2_EDTA as stabilizer (BD, USA) and for serum separation, blood was collected in 8.5 mL Vacutainer Serum Separation plastic tubes which contain silica particles as a clot activator and a gel which forms a barrier between the serum and the clot after centrifugation (BD, USA). Up to 50 mL blood (~22 mL plasma) were collected from each volunteer for the time-course and serum/plasma comparison experiments, and 80 mL blood (~35 mL plasma) were collected from each of 5 healthy female volunteer donors (mean age 58 years, range 53 – 72 years) for methylation enrichment and Illumina Next-Generation Sequencing.

### Blood storage time-course

For the blood storage time-course studies, blood was stored for 4 hrs, 8 hrs, 24 hrs or 48 hrs after collection at 4°C prior to separation of plasma. Control tubes were processed immediately after collection. The time-course experiment was carried out 3 times using blood from separate donors. For methylation enrichment and sequencing studies, blood was stored for 6 hrs at 4°C prior to plasma separation. At the appropriate time-point, the blood tubes were centrifuged for 10 mins at 1370 g at 4°C in a Rotanta 460R benchtop centrifuge (Hettich, Germany). The plasma was carefully transferred into fresh 15 mL or 50 mL tubes (Corning, USA), and centrifuged again as above to remove any remaining cell debris. Plasma was stored at -70°C until DNA extraction.

### Comparison of plasma and serum

In order to compare the DNA extracted from plasma and from serum, blood was collected in K_2_EDTA tubes for plasma separation and in clot activator containing tubes for serum separation as described above. In addition, each type of tube was pre-loaded with 70 ng of purified genomic DNA (Roche) prior to blood collection. Both types of tube were incubated for 30 mins at room temperature to allow clot formation in the serum samples, and then centrifuged as described above. Plasma and serum were transferred into new tubes and centrifuged again as described to remove any remaining cell debris. DNA was extracted from the total volume of plasma or serum obtained from each tube (~4 mL of plasma and ~3 mL of serum) using the Circulating Nucleic Acids Kit (Qiagen) according to manufacturer’s instructions, and extracted DNA was visualized on a 1.5% TAE agarose gel post stained with Gel Red stain (Biotium). DNA quantitation by qPCR was carried out in triplicate for matched plasma and serum from 3 separate donors, whereas genomic DNA spiking and DNA agarose gel visualization was carried in duplicate using blood from 2 separate donors.

### DNA extraction

For fcDNA extraction from small volumes of plasma (200 μL), used for time-course DNA quantitation, the QIAamp MinElute Virus Spin Kit was used following manufacturer’s instructions. For fcDNA extraction from larger volumes of plasma (≥4 mL up to 35 mL), used for gel visualization of fcDNA, methylation enrichment, and Next-Generation Sequencing, the Circulating Nucleic Acids Kit (Qiagen) was used, with some modification to the manufacturer’s protocol. Briefly, plasma was combined with proportionately scaled volumes of proteinase K and ACL buffer. Scaled quantities of carrier RNA added to the samples were capped at 5 μg per sample in order to minimize interference in downstream steps. The samples were incubated at 60°C for 45 mins to compensate for the slower heating of larger volumes. A scaled volume of Buffer ACB was added to the digested samples and they were incubated for 5 mins on ice, and then applied in batches to the QIAamp mini column via the tube extender. Where the total volume of digested sample was ≤17.5 mL the sample was processed using a single column. Samples >17.5 mL were split across 2 columns and processed in parallel. Once all the lysate had been drawn through, the column was washed twice with 700 μL of Buffer ACW1, then once with 750 μL of Buffer ACW2. All further steps were carried out as specified in the manufacturer’s protocols, except for the 56°C incubation to dry the columns, which was reduced from 10 mins to 5 mins. The samples were eluted in 50 μL of AVE Buffer followed by a second elution of 30 μL, and a pooling of the separate elutions. Purified DNA samples were stored at -80°C until further use.

fcDNA samples (1 μL) were analyzed for size distribution using the High Sensitivity DNA Chip (Agilent Technologies) on an Agilent Bioanalyzer according to manufacturer’s instructions, or on a 1.5% agarose TAE gel post stained with Gel Red stain (Biotium).

### Methylation enrichment

Methylated DNA sequences were isolated using the MethylMiner kit (Invitrogen). Prior to commencing experiments on fcDNA, we evaluated the performance of the methylation enrichment protocol with low (100 ng) DNA sample amounts, and developed a modified high-stringency protocol to limit the amount of non-specific DNA binding. Briefly, in the high-stringency protocol, methyl-binding protein (MBD2) was coupled to the beads following kit instructions; however, only 1 μL of beads per sample was used. Once bead coupling was complete, all subsequent wash steps were carried out in 300 mM NaCl 1X High Stringency Wash buffer (HSW buffer), instead of 1X Wash/Bind buffer. 4X HSW buffer was made up by combining the supplied High Salt buffer with the 5X Wash/Bind buffer in a 1:2.67 volume:volume ratio. To capture methylated DNA 150 μL of DNA was mixed with 50 μL of 4X HSW buffer and this solution was used to directly resuspend the washed MBD-protein coupled beads. The mixture was incubated on a rotor at 4°C overnight, the unbound DNA was removed, and the beads were washed with HSW buffer 3 times. The captured DNA was eluted from the beads in a single high salt elution step, ethanol precipitated as per kit instructions, resuspended in 35 μL H_2_O, and stored at -80°C until further analysis. For samples processed using the standard protocol, the manufacturer’s instructions were followed without modification. The two protocols were evaluated side by side with 100 ng and 400 ng DNA from human peripheral blood mononuclear cells (PBMCs), and from an SSSI-treated fully methylated control (Millipore). Yield was determined by qPCR of the *SFTA3* promoter sequence as described below. Based on the data from protocol evaluation, the high-stringency protocol was used to isolate methylated sequences from fcDNA samples.

Blood from 5 separate donors was used for the fcDNA methylation enrichment followed by Next-Generation Sequencing experiment. fcDNA was subjected to methylation enrichment without any additional fragmentation, while DNA fully methylated *in vitro* with SSSI enzyme (Millipore), which was used as a methylation positive control, was fragmented using a Branson Digital Sonifier Model 450 probe sonicator (Branson Ultrasonics Corporation, USA) to a size range of around 100–500 bases. 100 ng of the SSSI methylation positive control DNA was processed in parallel with the fcDNA samples.

### PCR DNA quantification

Quantitative PCR was carried out on a Corbett RotorGene 2000 machine (Sydney, Australia) in a 20 μL reaction volume containing 0.6 U Taq Polymerase (Roche), 1X PCR reaction buffer (Roche), 0.2 mM dNTP (Roche), 0.4 μM of each primer, 3% DMSO, and SYBR green I (Invitrogen) at a final dilution of 1/25000. Following initial denaturation for 8 minutes at 95°C, the PCR cycles were as follows: 10 sec at 95°C, annealing for 45 sec at 60°C, extension for 30 sec at 72°C, with a data acquisition step at the end of the extension. Primers targeting the promoter region of the *SFN1* gene (F – GCCAAGAGCAGGAGAGACAC; R – TTGGCCTTCTGGATCAGACT) or the *SFTA3* gene (F –AGCCTCTTTCTTGCCATCAA; R –ACGCTTCAGATTGCGTTCTA) were used for data in the main figures. These genes were selected as we have found these reactions to be particularly robust and sensitive. In addition, the *SFTA3* promoter is found to be unmethylated in PBMCs (data not shown), hence this assay is suitable for comparing yields of unmethylated (PBMC) and *in vitro* methylated (SSSI treated) DNA. For the DNA quantitation shown in Additional file 1: Figure S1, TaqMan RNAse P primers (Invitrogen) were used following manufacturer’s instructions. For all PCR quantitation assays, DNA concentration was determined by comparison against a standard curve of genomic DNA.

### Next-Generation Sequencing and analysis

Next-Generation Sequencing to generate 50-base, single-end reads was carried out on the Illumina HiSeq2000 platform (The Ramaciotti Centre for Gene Analysis, UNSW). The sequencing library was prepared using the ChIP-Seq DNA Sample Preparation Kit from Illumina, following the manufacturer’s instructions from the “Preparing Samples for ChIP Sequencing of DNA” booklet (2007). A modification was introduced at the library size restriction step with the use of the Pippin Prep (Sage Science) to collect a size range corresponding to 180 ± 50 base DNA fragments in order to be certain of capturing the 180 base fcDNA band, taking into account the additional length of the adapters and primers which added 92 bases to the fragments. Successful library purification was verified by running the DNA on an Agilent 2100 Bioanalyzer High Sensitivity Chip in combination with fluorescence measurements by the QuBit fluorometer (Invitrogen) to check for recovery.

Basic quality control checks and % GC content calculations were carried out using FastQC (http://www.bioinformatics.bbsrc.ac.uk/projects/fastqc) and sequences were mapped to the Hg19 version of the human genome using Bowtie [19], allowing for up to 3 mismatches. Uniquely aligned reads were used in subsequent analyses. Visualization and analysis were carried out in IGV [20] and Galaxy (https://usegalaxy.org/). Peaks of methylated regions were called via the MACS algorithm [21] and library fragment size was estimated using Homer DNA analysis software [22].

### Clonal bisulphite sequencing

Primers (Additional file 1: Table S1) were designed to amplify both methylated and unmethylated bisulphite converted DNA. PCR conditions for unbiased and specific amplification were determined using different MgCl_2_ gradient and PCR annealing temperatures on DNA from the double knock-out cell line HCT116 (unmethylated control) and the same cell line DNA enzymatically methylated *in vitro* (fully methylated control) (Zymo). Bisulphite conversion was carried out on fcDNA donor sample 5 using the Epitect kit (Qiagen). Having identified the appropriate PCR conditions, 150 ng total fcDNA was bisulphite converted using the “Small Amounts of Fragmented DNA” protocol from the Epitect Kit, PCR amplified, and cloned into the pCR2.1 vector using the TA Cloning Kit (Invitrogen) following manufacturer’s instructions. Bacterial colonies with positive clones were chosen using blue-white selection on IPTG/X-gal plates. Sanger sequencing was carried on 12–13 clones for each amplicon (Additional file 1: Table S1), using standard protocols.

## Results and discussion

fcDNA holds great promise as a non-invasive source for real-time disease markers, and thus has multiple useful clinical applications. Because it is typically found at very low concentrations in plasma, successful downstream analysis requires prompt sample processing and standardized protocols that can accommodate very small amounts of input material. However, there are currently no definitive standards for blood collection and processing, and fcDNA extraction. We sought to standardize methodology for sample storage, processing and scaling, to recover optimal fcDNA quantities and quality for downstream whole-genome analysis.

### Comparison of plasma and serum

**Figure 1:**
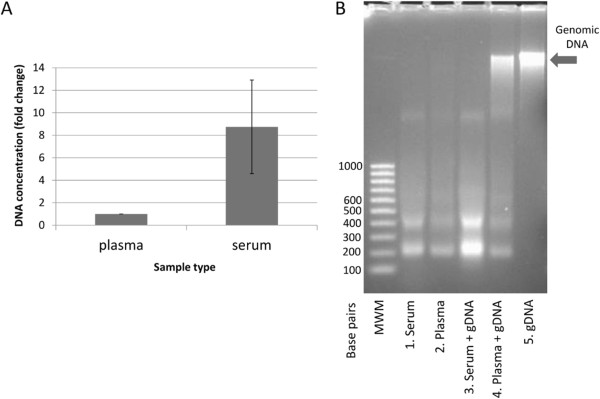
**Comparison of fcDNA isolation from plasma and serum. (A)** PCR quantitation of fcDNA obtained from 200 μL plasma and serum samples. Bars represent the average concentration fold change in matched serum and plasma samples obtained from 3 separate donors ± SD. **(B)** Lane 1: fcDNA isolated from 4 mL serum; Lane 2: fcDNA isolated from 4 mL plasma; Lane 3: genomic DNA spiked into serum sample prior to fcDNA processing; Lane 4: genomic DNA spiked into plasma sample prior to fcDNA processing; Lane 5: genomic DNA. Arrow: high molecular weight genomic DNA; MWM: molecular weight marker.

To date, most publications describing fcDNA studies have utilized either serum or plasma as their DNA source, with higher yields of fcDNA being reported from serum than from plasma [3, 4, 23–25]. However, it is currently unclear whether the quality of fcDNA extracted from serum *versus* plasma is comparable. To address this, we compared yields and visualization of fcDNA extracted from equivalent volumes (4 mL) of serum or plasma from the same donors. A higher yield of DNA was observed from serum (32.7 ± 19.9 ng/mL) than from plasma (3.6 ± 0.5 ng/mL) (Figure 1A). The increased yield from serum has been postulated to be due to contamination with genomic DNA released from leukocytes which lyse during the clotting and centrifugation procedures undertaken in serum collection [25]. In order to determine whether high molecular weight genomic DNA was present in our samples, we visualized the plasma- and serum-derived fcDNA on an agarose gel. The fcDNA appeared as a band at ~180 base pairs, and a minor band between 300–400 base pairs (Figure 1B, Lanes 1 and 2), reflecting the fcDNA fragments, similar to previous reports [1, 2]. We did not detect a band corresponding to genomic DNA in either sample type. To eliminate the possibility that high molecular weight DNA released during blood processing was not detectable on a gel because it was degraded by active nucleases, serum and plasma blood collection tubes were pre-loaded with purified leukocyte genomic DNA prior to sample collection. Blood was then collected into the pre-loaded tubes, and plasma or serum was separated. The fcDNA was then extracted and visualized on an agarose gel (Figure 1B, Lanes 3 and 4). The genomic DNA that we had pre-loaded in the plasma tube was recovered and visible as a high-molecular weight band co-migrating with purified genomic DNA (Figure 1B, Lane 5), while no corresponding band was visible in the serum isolated from the pre-loaded tube.

This finding that we were unable to recover spiked genomic DNA from serum suggests that DNA released from leukocytes during clot formation and centrifugation could be degraded by the DNAses that are active in serum, such as DNAse1 and DNAse1l3 [26]. As genomic DNA spiked into plasma tubes is not degraded, the responsible DNAse is likely inhibited by the EDTA present in the plasma tube to prevent blood clotting. We also note that there is an apparent increase in the 180 bp band in the serum sample spiked with genomic DNA (Figure 1B, Lane 3). This increase was consistently observed across repeat experiments using blood samples from different donors (data not shown). As it is unlikely that the spiked nucleosome-free genomic DNA would be degraded to regular sized fragments, this increase may represent additional leukocyte lysis stimulated by the presence of naked DNA in the sample; however a more detailed investigation of this point is beyond the scope of this study.

### Effect of blood storage times on plasma and serum DNA concentration

**Figure 2:**
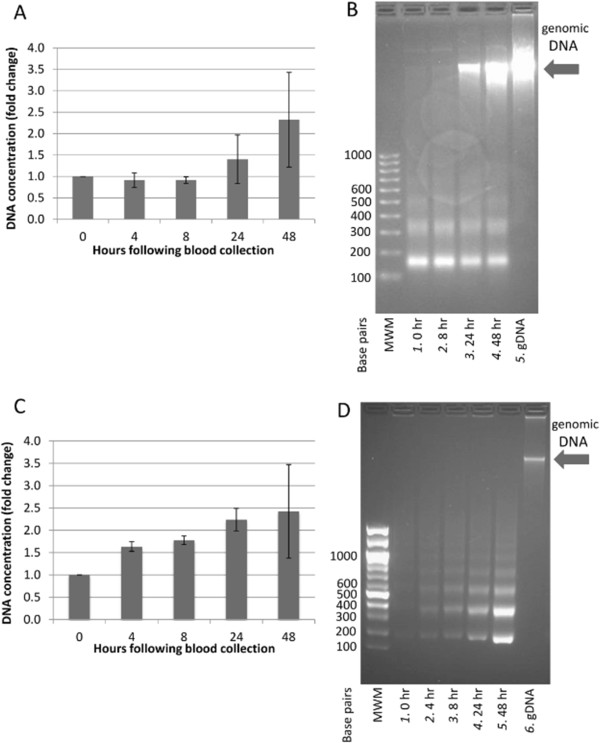
**Contamination of fcDNA with genomic DNA during blood storage.** Concentration change of DNA extracted from plasma **(A)** or serum **(C)** measured by PCR at 0 hr, 4 hr, 8 hr, 24 hr and 48 hr following blood collection. Each bar represents the average concentration fold change of triplicate **(A)** or duplicate **(C)** experiments ± SD. **(B)** fcDNA isolated from 4 mL plasma immediately (Lane 1), 8 hrs (Lane 2), 24 hrs (Lane 3) and 48 hrs (Lane 4) following blood collection. Lane 5: genomic DNA. **(D)** fcDNA isolated from 3 mL serum immediately (Lane 1), 4 hrs (Lane 2), 8 hrs (Lane 3), 24 hrs (Lane 4) and 48 hrs (Lane 5) following blood collection. Lane 6: genomic DNA. Arrow: High molecular weight genomic DNA; MWM: molecular weight marker.

As research laboratories are frequently at some distance from the clinical facilities where biospecimens are collected from patients, leading to delays between sample collection and processing, we sought to determine an acceptable time interval between blood collection and plasma or serum separation prior to fcDNA extraction. To examine the effects of blood storage times on the cell free DNA content, we collected blood from healthy volunteers and either processed it immediately, or stored it at 4°C for 4 hrs, 8 hrs, 24 hrs or 48 hrs prior to plasma or serum separation. fcDNA was then extracted from 4 mL plasma or 3 mL of serum, quantitated by qPCR and examined on a gel to determine its stability over time (Figure 2). We observed no change in DNA content in plasma up to 8 hrs (range 4.4-4.9 ± 1.3 ng/mL); however DNA concentrations in the plasma increased steadily at 24 (6.5 ± 2.2 ng/mL) and 48 hrs (10.8 ± 4.5 ng/mL) after storage (Figure 2A). In contrast, serum DNA concentration was increased by 4 hours, and continued to increase as the blood was stored over time (Figure 2C). In order to examine the fcDNA and determine what may be contributing to the increased concentrations during storage, the samples were visualized on an agarose gel (Figure 2B and D). At each time point examined, the plasma samples contained a strong band at ~180 bp, and a minor band between 300 bp and 400 bp (Figure 2B, Lanes 1–4), representing the fcDNA fragments, while the 24 hr and 48 hr time points also contained a high molecular weight DNA band, which appeared in parallel to the increase in DNA concentration shown in Figure 2A. As this high molecular weight band co-migrates with purified genomic DNA (Figure 2B, Lane 5), it most likely represents DNA released from leukocytes which lysed during blood storage. The gel of DNA extracted from the serum samples also shows a clear increase in the DNA with increasing blood storage times, but no high molecular weight band is apparent; rather, there is an increase in laddered DNA (Figure 2D, Lanes 1–5). This suggests that during blood clotting and storage, leukocytes either undergo apopotosis, producing the characteristic DNA cleavage pattern, or that serum active nucleases cleave DNA released from lysed leukocytes inter-nucleasomally.

Although storage of blood prior to plasma and serum separation introduces DNA into the sample which differs in size depending on the sample type, the most likely source of this DNA in either case is lysis of leukocytes during storage and release of their DNA into the blood, thereby contaminating any fcDNA present in the sample. Therefore, if fcDNA studies intend to investigate shed cell-free DNA found in the circulation, we recommend that the maximum time that blood samples should be stored prior to plasma separation is 8 hrs, whereas serum samples should be processed immediately to prevent contamination with genomic DNA. This is in agreement with previous reports of artifacts introduced through storage of blood samples prior to processing [23]. Furthermore, we note that lack of a high molecular weight band in serum is not indicative of absence of genomic DNA contamination, and previous work using male white blood cells spiked into female derived blood samples has determined that leukocytes lyse during the process of clotting [25]. Therefore, in order to avoid potential genomic DNA contamination in fcDNA studies, plasma should be the preferred source for fcDNA extraction.

### fcDNA purification from large plasma volumes

Once we developed optimized protocols for blood collection and fcDNA processing, we undertook a study to capture and sequence methylated fcDNA from 5 volunteer blood donors. In order to isolate sufficient fcDNA from blood for methylation enrichment and Next-Generation Sequencing, fcDNA must be extracted from large volumes of plasma, indicating a need to scale up standard DNA isolation protocols while avoiding excessive dilution. The QIAamp Circulating Nucleic Acids kit (Qiagen) specifies plasma volumes of up to 5 mL; however, for whole-genome analysis of methylated fcDNA, which represents a small fraction of total fcDNA, an input volume of at least 35 mL is necessary. For our studies, we modified the standard Qiagen protocol for increased plasma volumes, including proportional scaling up of the proteinase K and ACL buffer. At the column binding step this volume was split across two columns (17.5 mL each), and the eluted DNA for each sample was pooled. While the time for sample processing was increased, we did not encounter any issues with column blockage at these larger volumes. A pilot experiment was carried out to show that proportional yield did not decrease with scaling up to a volume of 17.5 mL (Additional file 1: Figure S1).

### DNA isolation and methylation enrichment from modified MethylMiner protocol

**Figure 3:**
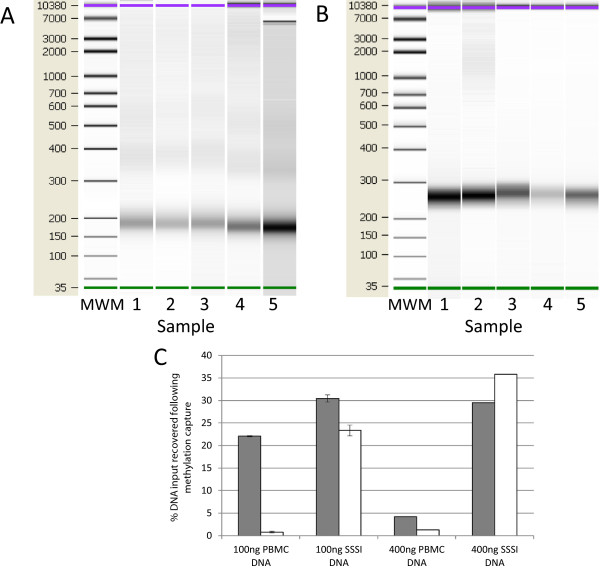
**Size distribution of fcDNA in 5 control samples and following fcDNA library construction (A and B) and DNA recovery following MBD-capture (C).** Agilent Bioanalyzer chip with fcDNA isolated from 5 control subjects prior to **(A)** and following **(B)** methylation enrichment and library preparation. Increase in molecular weight reflects successful adapter ligation. MWM: molecular weight marker. DNA recovery following MBD-capture **(C)**. Recovery of DNA (based on % of input DNA) as quantitated by qPCR following the standard (grey bars) or our modified (white bars) MBD-capture protocol. *SFTA3* promoter is unmethylated in PBMC DNA and methylated in the methylation positive control SSSI. At low DNA inputs (100 ng), *SFTA3* is recovered from both PBMC and SSSI DNA, suggesting high background MBD binding and low methylation enrichment. With our modified protocol, *SFTA3* is minimally recovered from PBMC DNA (unmethylated) but is recovered from SSSI DNA (methylated), even at low DNA inputs. Data is presented as % DNA recovery ± range.

The plasma concentration of fcDNA isolated from 35 mL of plasma in 5 control subjects ranged from 6.9 – 10.7 ng/ml plasma (Table 1), comparable with concentrations previously reported for healthy individuals [8]. The size distribution of the fcDNA was visualized on an Agilent Bioanalyzer chip. As expected, we observed a very strong band at 180 base pairs, with fainter, more diffuse bands at 300–400 base pairs (Figure 3A). The next step involved application of a methyl-binding protein capture protocol (MBD-cap) to capture methylated fragments from total fcDNA isolated from plasma. Because our starting fcDNA concentrations were low (Table 1), we investigated the efficiency of MBD-capture to ensure specific enrichment of methylated fragments. Using peripheral blood mononuclear cell (PBMC) DNA and methylation-positive SSSI DNA, we quantitated DNA recovery by qPCR of the *SFTA3* promoter region following MBD-capture of low (100 ng) and standard (400 ng) DNA inputs (Figure 3C), using both the standard capture protocol and our modified protocol. As *SFTA3* is unmethylated in PBMC and methylated in SSSI DNA, we expect low recovery of *SFTA3* from PBMC DNA and high recovery from SSSI DNA. Our studies determined that with low DNA input, the standard MBD-cap protocol results in high levels of background binding of DNA to the MBD-linked beads, and thus only minor enrichment of methylated sequences (Figure 3C, 100 ng, grey bars), whereas with standard DNA inputs, background binding is proportionately significantly decreased and methylation enrichment reaches at least 7-fold (Figure 3C, 400 ng, grey bars). Because our total fcDNA input was ~50 ng (Table 1), we modified the standard protocol to minimize the non-specific DNA binding at low inputs. Specifically, we decreased the volume of beads used from 10 μL to 1 μL, and increased the stringency of the Bind/Wash buffer to 300 mM NaCl. Using this modified MBD-cap protocol, background binding of DNA to the MBD-linked beads was minimal even at low DNA inputs, with methylation enrichment reaching 30-fold (Figure 3C, white bars). Thus, our modified MBD-cap protocol allows for low background binding and specific enrichment of methylated fragments from low DNA inputs. This modified protocol was then applied to our fcDNA samples, and the percent recovery of methylated DNA following our modified MBD-capture protocol ranged from 10.2-14.9% (Table 1), comparing well with the approximately 7% recovery previously described in the literature [17]. There are currently a number of methylation-capture kits available (recently evaluated for performance in [27]), which may perform at various levels based on the biological sample input.

**Table 1:** **Concentration of fcDNA in 5 control subjects and DNA quantitation at consecutive stages of sample processing**

Sample	Plasma fcDNA concentration (ng/mL)	MethylMiner input (ng)	MethylMiner recovery (ng) (%)	Illumina NGS* input for library generation (ng)	Amount of library generated (ng)
1	6.9	48.7	5.04 (10.4)	4	464
2	7.3	49.6	5.04 (10.2)	4	245
3	7.8	49.5	5.73 (11.6)	4	314
4	9.9	42.2	6.28 (14.9)	4	39
5	10.7	43.2	5.32 (12.3)	4	222

*NGS = Next-Generation Sequencing.

### Library preparation

The methylation-enriched fcDNA samples were then prepared for Illumina Next-Generation Sequencing. MBD-capture followed by sequencing is conceptually similar to ChIP-Seq where fragments of DNA are captured by specific binding to an antibody directed against a transcription factor; however in place of the antibody a domain of the methyl binding protein MBD2 is used. Thus, we applied a modified version of the Illumina protocol “Preparing Samples for ChIP Sequencing of DNA” to generate the sequencing library. Because our fcDNA input was less than half of the minimum DNA input required by Illumina, we took advantage of the discrete size of fcDNA to minimize sample loss during library preparation. In the ChIP-Seq protocol, DNA is fragmented by sonication, producing fragments with a range of sizes that run as a broad band/smear on an agarose gel. During library preparation, fragments are then size-restricted by excising a gel slice within the desired size range and re-purifying the DNA from this slice. We reasoned that a significant loss of input DNA occurs at the size restriction step, as most of the DNA is left behind in the agarose gel since it falls outside the appropriate size range. fcDNA samples would not be subjected to this loss, since the DNA is of a uniform size, and runs predominantly as a single band which could be recovered in its entirety from within the gel slice. Because of this advantage, we postulated that libraries of good complexity could still be generated from fcDNA inputs considerably less than 10 ng minimum specified by the Illumina protocol. This approach restricts the DNA captured to the lowest molecular weight band, and excludes high molecular weight DNA; however, previously reported whole genome sequencing of the low molecular weight fraction of fcDNA was able to clearly distinguish between control and cancer samples, indicating that this fraction contains sufficient DNA to be detectable by NGS [14].

We used an input amount of 4 ng fcDNA for the protocol, and recovered 39–464 ng following library preparation (Table 1). The prepared libraries were visualized on a Bioanalyzer Chip (Figure 3B). The size of the libraries ranged from 266 bases to 269 bases, which is in good agreement with the expected size of 272 bases (180 base DNA fragments plus 92 base adapters), indicating that we were able to obtain good quality and complete libraries with less than half of the required amounts of DNA. The length of the sequenced fragments, excluding adapters, was further validated using the Homer tag autocorrelation function [22], in which the position of each read is calculated relative to every other read on the same chromosome. This analysis revealed an average sequence fragment length estimate of 170 bases, ranging between 165 and 173 bases across the 5 sequenced samples (data not shown), consistent with the size of the fcDNA observed on the Bioanalyzer chip.

### Next-Generation Sequencing results and quality control

Our 5 fcDNA samples were then submitted for 50-base, single-end Next-Generation Sequencing. Read numbers are presented in Table 2. We obtained 37×10^6^ to 86×10^6^ unique mappable reads per sample, which is well above the 10x10^6^ unique mappable reads per biological replicate suggested as a minimum for ChIP-seq experiments in mammalian cells by the ENCODE consortium [28]. For all samples, unique mappable reads represented more than 50% of the total mappable reads, indicating that despite the relatively low DNA input amounts, a good level of library complexity was achieved [29].

**Table 2:** **Descriptions of Next-Generation Sequencing read numbers**

Sample	Total reads ^1^	Unaligned ^2^	Multiple site aligned ^3^	Single site aligned ^4^	Unique ^5^	% Unique
1	197,921,529	16,066,294	44,319,996	137,535,239	76,590,535	55.7
2	199,302,552	15,885,135	43,676,951	139,740,466	77,836,232	55.7
3	108,955,707	8,729,606	26,671,313	73,554,788	43,869,724	59.6
4	109,132,802	11,042,561	24,138,010	73,952,231	37,270,330	50.4
5	200,639,214	15,558,416	44,708,970	140,371,828	85,538,368	60.9

^1^Total reads obtained.

^2^Reads which could not be aligned to a site within the human genome.

^3^Reads which could not be accurately mapped since they aligned to multiple sites within the human genome.

^4^Reads which aligned to a single site within the human genome.

^5^Unique reads which aligned to a single site within the human genome.

^6^Unique reads as a percentage of the reads which could be aligned at a single site within the genome.

**Figure 4:**
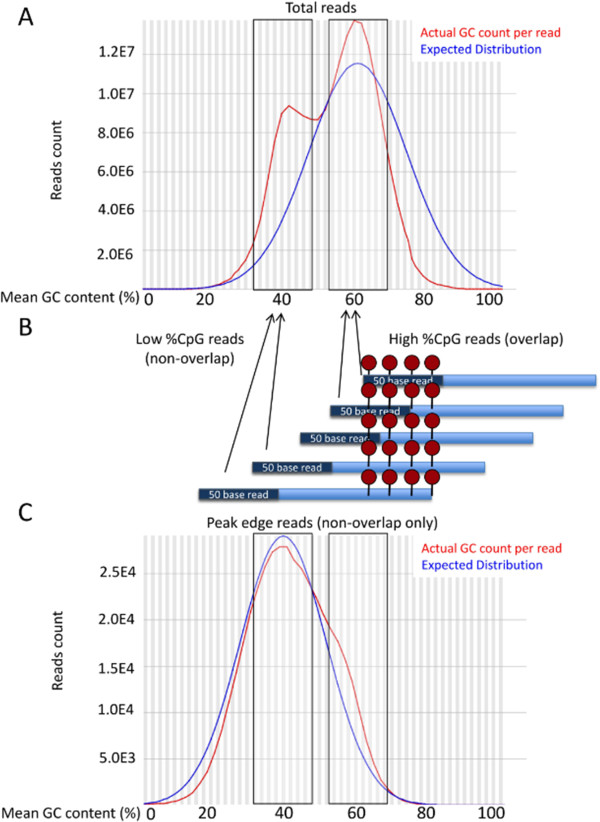
**% GC distribution of sequenced reads. (A)** Expected (blue line) and actual (red line) % GC content present in total sequenced reads. Representative reads from a single sample are shown. **(B)** Elucidation of source of high and low % GC peaks in the sequencing reads. Model depicts captured fcDNA fragments (blue lines with sequenced 50 base reads shown on ends) containing methylated CpG sites which allow for capture (red circles). Arrows indicate the contribution of each read to the low % GC peak (methylated CpG sites are located in the middle of the fragment and thus fall outside 50 base read) and the high % GC peak (methylated CpG sites are located near the edge of the fragment and thus contained within 50 base read). **(C)** Expected (blue line) and actual (red line) % GC content present in non-overlapping sequenced reads. Representative reads from a single sample are shown.

Quality control checks were carried out on obtained reads using FastQC, and showed that base quality scores were consistently high (>28) for all samples (Additional file 1: Figure S2). The quality control analysis also revealed that we did not observe the expected normal distribution of % GC content across 50-base sequencing reads (Figure 4A, blue line, centered at 59%). Instead we observed a bimodal distribution of % GC content, with one (high) peak centered on 59% GC, and the other (low) peak centered on 39% GC (Figure 4A, red line). We postulated that this bimodal distribution reflects sequenced reads that either overlap with the CpG sites that allowed MBD capture in each fragment (corresponding to high % GC reads), or that fall outside the CpG sites that led to the capture of the DNA fragment (corresponding to low % GC reads). Because the sequencing process starts at one end of the fcDNA fragment and reads the 50 bases adjacent to that end, sequence reads will either overlap with methylated CpG sites if they are located near the end of fcDNA fragments, thus displaying a high % GC peak, or they will sequence outside methylated CpG sites if they are located in the middle of fcDNA fragments, thus displaying a low % GC peak (Figure 4B). To test this model, we used FastQC to calculate the expected and actual % GC content of sequenced reads positioned at the outer edge of called peaks of methylated regions, which would not overlap with the methylated CpG sites allowing for capture of the fcDNA fragment. The GC plots generated from these reads correspond to the low GC content peak (39%) of the bimodal distribution, whereas the high GC content peak (59%) is no longer present (Figure 4C). These results support the notion that the bimodal % GC distribution seen across all 50-base sequencing reads is driven by the position of methylated CpG sites of each captured DNA fragment either overlapping the sequenced end or falling outside of it.

### Sequencing validation

**Figure 5:**
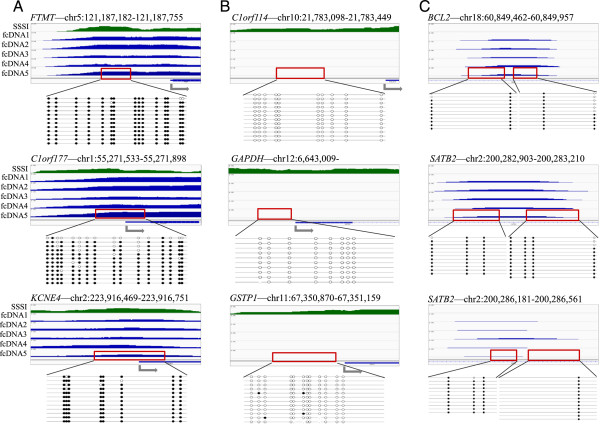
**Clonal bisulphite sequencing of fcDNA from sample 5. (A)** Validation of 3 promoter regions found to be methylated in SSSI positive control (green) and in fcDNA (blue). **(B)** Validation of 3 promoter regions found to be methylated SSSI positive control (green) but not in fcDNA (blue). **(C)** Validation of 3 loci found to be unmethylated in SSSI positive control (green) but methylated in fcDNA (blue). Closed circles indicate methylated CpGs as determined by clonal bisulphite sequencing; open circles indicate unmethylated CpGs as determined by clonal bisulphite sequencing; grey arrows indicate transcriptional start sites; red rectangles indicate the regions sequenced for validation.

In order to validate our sequencing results, and verify that our sequenced fragments specifically represented methylated fcDNA regions captured by binding to the MBD2 protein rather than DNA which had non-specifically bound to the bead matrix or the plasticware, we carried out bisulphite conversion and clonal sequencing on selected regions in pre-methylation-enriched fcDNA from sample 5. We selected 3 regions that appeared methylated in the fcDNA and in the methylation positive control sample (*FTMT*, *C1orf177* and *KCNE4* promoter regions) (Figure 5A), 3 regions that appeared unmethylated in the fcDNA and methylated in the positive control sample (*C10orf114*, *GAPDH* and *GSTP1* promoter regions) (Figure 5B), and 3 regions that appeared methylated in the fcDNA despite no signal being observed in the methylation positive control (*BCL2* and *SATB2* gene body regions) (Figure 5C). DNA was bisulphite converted, PCR-amplified, cloned, and 12–13 clones were sequenced for each region. In all 9 examined genomic regions, the clonal bisulphite sequencing results were concordant with the methylation results from the MBD-capture and Next-Generation Sequencing. We postulate that the lack of signal in the methylation positive control shown in Figure 5C is due to either incomplete *in vitro* enzymatic methylation, or to regions with sparse CpGs being out-competed from the MBD-binding sites by the abundant dense methylation in the remainder of the positive control DNA. In either case, the clonal bisulphite sequencing results demonstrate that the sequencing reads obtained for these genomic regions in the fcDNA samples do not represent false positives. These results provide further support that whole-genome analysis of even small amounts of fcDNA can provide high-quality, validated genomic data that strengthen the potential of fcDNA utility in clinical applications.

## Conclusions

While tissues and cell lines are readily amenable to methylated DNA capture and sequencing due to the relatively large amounts of starting material typically available, sequencing of the methylated fraction of fcDNA has not previously been reported, most likely due to the technical challenges presented by working with very small amounts of input DNA. We developed and applied modified protocols for plasma DNA extraction, methylated sequence enrichment and sequencing library construction, allowing us to obtain high numbers of good quality unique reads from methylated fcDNA samples. This approach allows in-depth genomic characterization made possible by Next-Generation Sequencing to be applied to tiny amounts of methylated fcDNA, for investigating biological mechanisms and developing diagnostic applications.

## Electronic supplementary material

Additional file 1: Table S1: Primer sequences and conditions used for unbiased amplification of methyated and unmethylated bisulphite-converted DNA. **Figure S1.** fcDNA quantitation by PCR. fcDNA concentration (ng) in 250 μl eluent from 5, 10 and 17.5 ml plasma samples. Each bar represents the average of duplicate experiments ± range. **Figure S2.** Sequence read quality for 5 fcDNA samples. Illumina sequence read quality in 5 fcDNA samples for each base pair (bp) position across the 50-bp reads. Q scores >28 (green section) are considered high-quality. (DOCX 281 KB)

## Abbreviations

fcDNA: Free circulating DNA
HSW: High-stringency wash buffer
MBD2: Methyl binding domain 2
NGS Next: Generation Sequencing
PBMC: Peripheral blood mononuclear cells.
