# Supplementary material for: Methylation-capture and Next-Generation Sequencing of free circulating DNA from human plasma
Source: BMC Genomics. 2014 Jun 15;15(1):476. doi: 10.1186/1471-2164-15-476 (PMC4078241; doi:10.1186/1471-2164-15-476)
Supplement: Supplementary file 1 — Additional file 1: Table S1: Primer sequences and conditions used for unbiased amplification of methyated and unmethylated bisulphite-converted DNA. Figure S1. fcDNA quantitation by PCR. fcDNA concentration (ng) in 250 μl eluent from 5, 10 and 17.5 ml plasma samples. Each bar represents the average of duplicate experiments ± range. Figure S2. Sequence read quality for 5 fcDNA samples. Illumina sequence read quality in 5 fcDNA samples for each base pair (bp) position across the 50-bp reads. Q scores >28 (green section) are considered high-quality. (DOCX 281 KB) [file 12864_2014_6172_MOESM1_ESM.docx]

Supplementary Table 1. Primer sequences and conditions used for unbiased amplification of methyated and unmethylated bisulphite-converted DNA.

|  | Region | Primer Sequences | MgCl2 | Annealing |  |
| --- | --- | --- | --- | --- | --- |
|  |  |  | [mM] | temperature [°C] |  |
|  | *FTMT* | Forward: GGTGGGGGIGAATGTTTA |  |  |  |
|  | promoter | Reverse: ACICCCCTTCTATTCCAAAA | 2.0 | 58.5 |  |
|  | *C1orf177* | Forward: AAGTTAAAAATTAGIGGGTTA |  |  |  |
|  | promoter | Reverse: ACTTTCCCTCATAACAAACCT | 2.0 | 54.6 |  |
|  | *KCNE4* | Forward: GAAAATTTGTAGTTTAATAATGGTT |  |  |  |
|  | promoter | Reverse: ACCCIACCAAAATTTTCAA | 2.0 | 54.5 |  |
|  | *C10orf114* | Forward: TTGAGTTTTAATATIGAGTTTTAATATGTG |  |  |  |
|  | promoter | Reverse: CCAAACTCCTTCCCAAACTTC | 2.0 | 55.8 |  |
|  | *GAPDH* | Forward: GTTIGGTGIGTGTTTAGTTG |  |  |  |
|  | promoter | Reverse: AACCAATCCCAACCCAAAAT | 2.0 | 58.4 |  |
|  | *GSTP1* | Forward: TTTGTGAAGIGGGTGTGTAA |  |  |  |
|  | promoter | Reverse: CAAATCCCCAACIAAACCTA | 2.0 | 58.3 |  |
|  | *BCL2* | Forward: GAAGAGGGTTTGGTTATTAGTT |  |  |  |
|  | gene body 1 | Reverse: TCCATCAAATATTAAAATCCTC | 2.5 | 54.4 |  |
|  | *BCL2* | Forward: GAGGATTTTAATATTTGATGGA |  |  |  |
|  | gene body 2 | Reverse: CACCCACCCTATAAACATCC | 1.5 | 52 |  |
|  | *SATB2* | Forward: AGAAGAGGAAATTGAGGTAGAGAA |  |  |  |
|  | gene body 1a | Reverse: TCTTTTACAATCTAATTCTAACTAAAAACA | 2.5 | 52 |  |
|  | *SATB2* | Forward: TGTAAAAGATATAGAATTAGAGGGTTA |  |  |  |
|  | gene body 1b | Reverse: CCTTTTAATTTTAAATATAATAAAAC | 3.0 | 52 |  |
|  | *SATB2* | Forward: TTGTTAATTTTTGGGTAAAGAAA |  |  |  |
|  | gene body 2a | Reverse: CCCACATTTTATTCATTTTCCTC | 2.5 | 52 |  |
|  | *SATB2* | Forward: GAGGAAAATGAATAAAATGTGGGTA |  |  |  |
|  | gene body 2b | Reverse: TTCATTTAACTTACTTTCTTACCTTT | 2.5 | 52 |  |

**Supplementary Figure Legends**

**Supplementary Figure 1: fcDNA quantitation by PCR.** fcDNA concentration (ng) in 250 µl

eluent from 5, 10 and 17.5 ml plasma samples. Each bar represents the average of duplicate

experiments ± range.

**Supplementary Figure 2: Sequence read quality for 5 fcDNA samples.** Illumina sequence read

quality in 5 fcDNA samples for each base pair (bp) position across the 50-bp reads. Q scores >28

(green section) are considered high-quality.

| **DNA concentration in 250** µ**l elution** |
| --- |

120

100

|  | 80 |  |
| --- | --- | --- |
| **(ng)** | 60 |  |
|  |  |  |

40

20

0

| 5mL | 10mL | 17.5mL |
| --- | --- | --- |


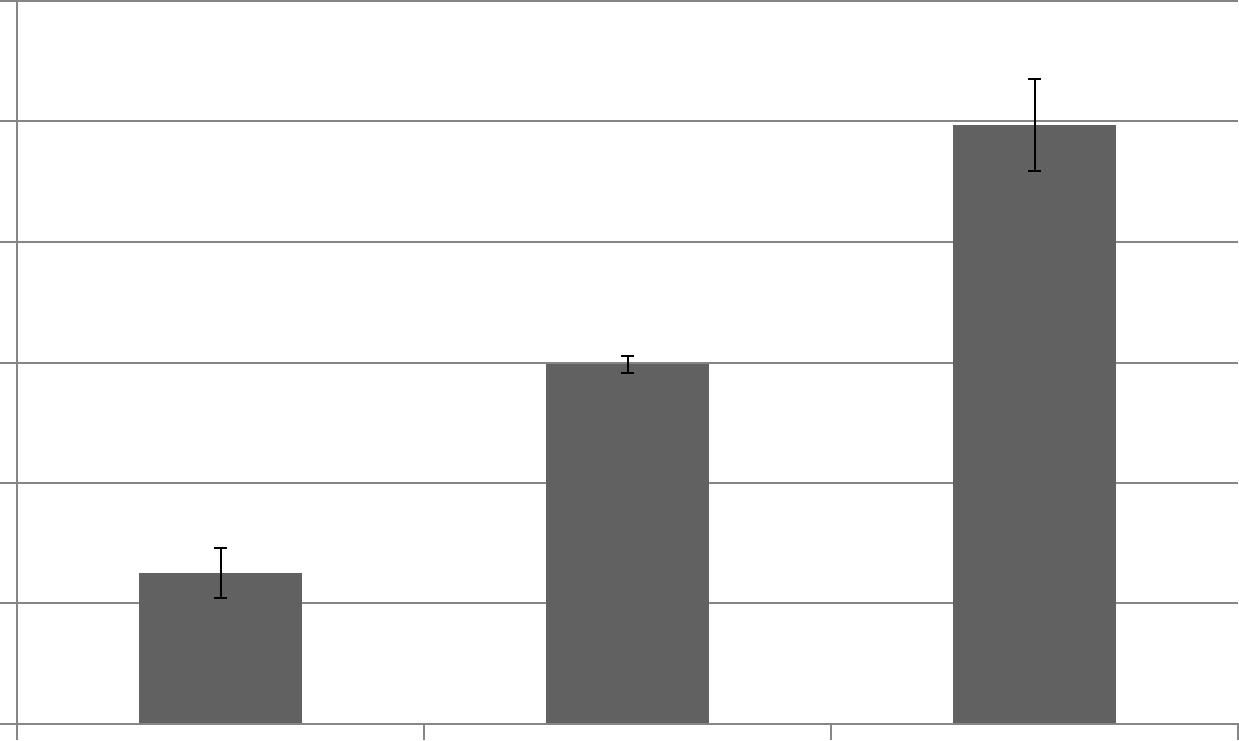


**plasma volume**

Supplementary Figure 1

Sample 1 Sample 2 Sample 3


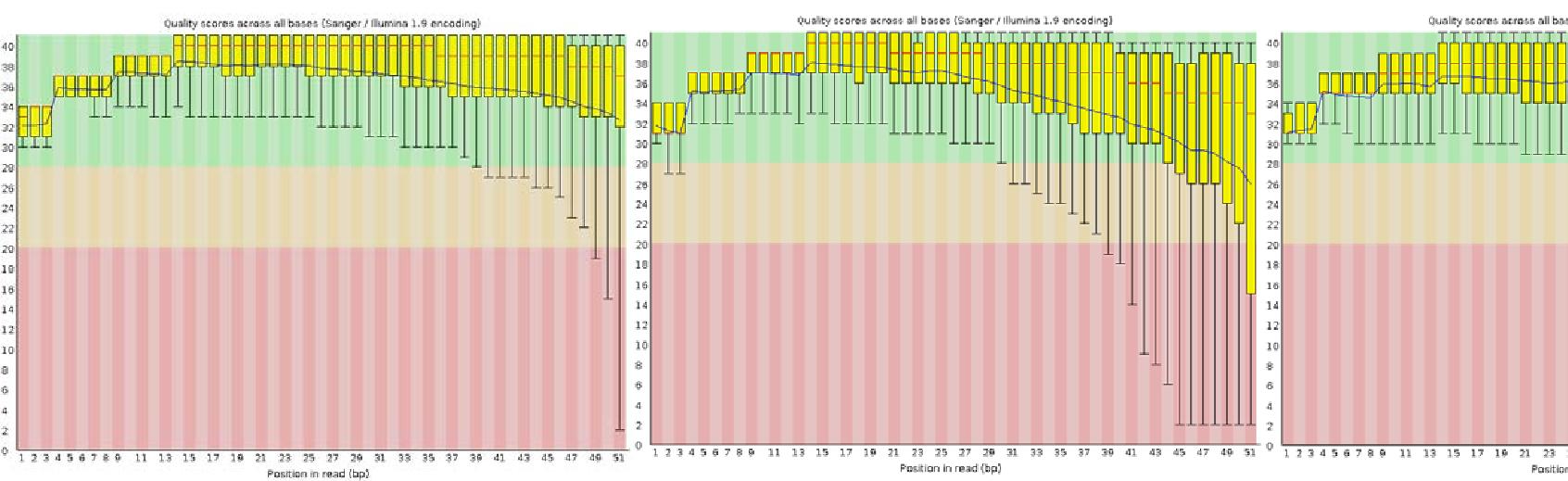


| Q score |
| --- |

Sample 4 Sample 5


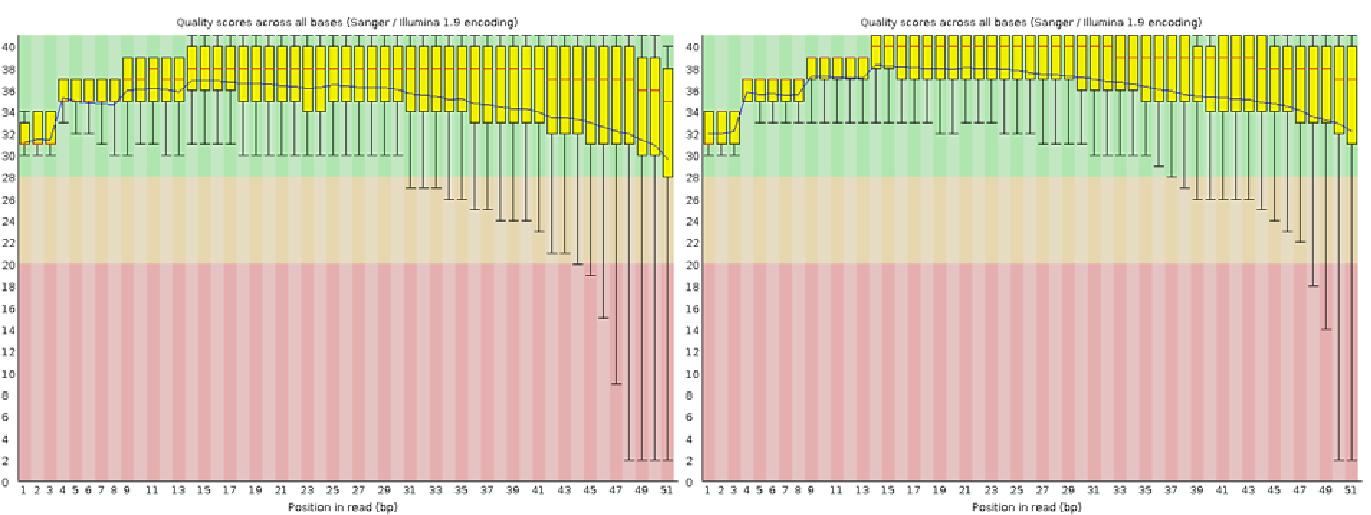


| Q score |
| --- |

Supplementary Figure 2
